# Adjusting the Crystallization of Tin Perovskites through Thiophene Additives for Improved Photovoltaic Stability

**DOI:** 10.1021/acsenergylett.4c01875

**Published:** 2024-10-08

**Authors:** Omar E. Solis, Miriam Mínguez-Avellán, Pablo F. Betancur, Raúl I. Sánchez- Alarcón, Isabelle Rodriguez, Juan P. Martínez-Pastor, Teresa S. Ripolles, Rafael Abargues, Pablo P. Boix

**Affiliations:** † Instituto de Ciencia de los Materiales de la Universidad de Valencia (ICMUV), 46980 Paterna, València Spain; ‡ Instituto de Tecnología Química, Universitat Politècnica València-Consejo Superior de Investigaciones Científicas, Av. dels Tarongers, 46022 València, Spain

## Abstract

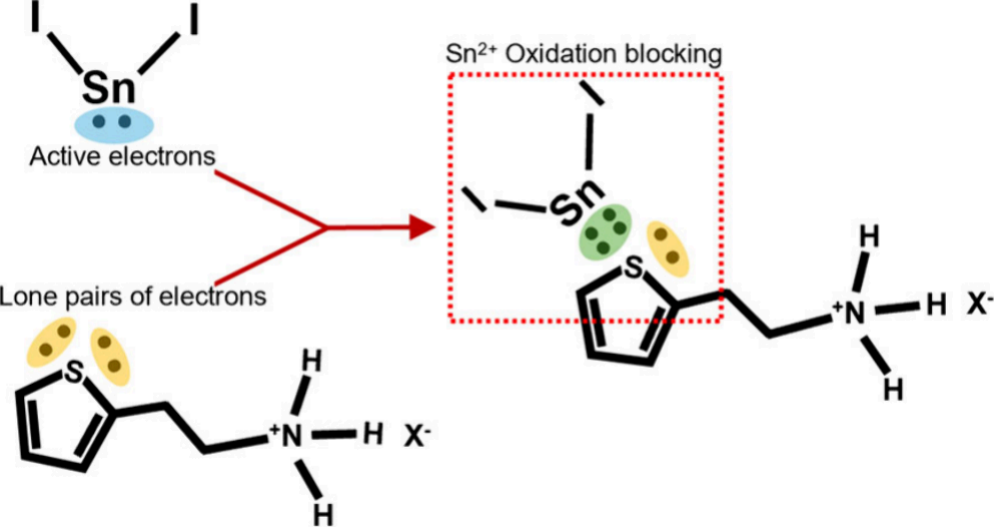

Tin-based perovskites (Sn-PVK) are promising lead-free alternatives for efficient photovoltaic technology, but they face challenges related to bulk and surface defects due to suboptimal crystallization and Sn^2+^ oxidation. Introducing thiophene-2-ethylammonium halides (TEAX, where X = I, Br, Cl) improves FASnI_3_ crystallization and reduces Sn^4+^ formation. This is achieved by adjusting the crystallization dynamics through the formation of a complex between S and Sn during the preparation of the precursor solution, which also inhibits Sn^2+^ oxidation in the resulting films. In solar cells, these additives boost power conversion efficiency (PCE) from 6.6% (without additives) to 9.4% (using TEABr), with further enhancement to 12% by adjusting selective contacts. The addition of TEAX also increases the Sn^2+^ content, outperforming control. Devices with TEABr maintained over 95% of their initial PCE after 2000 h in N_2_ under continuous operation with 1 sun simulated illumination.

Halide perovskite semiconductors’ excellent properties attract attention in optoelectronic research fields.^1−3^ They stand out as great candidates for technological revolutions in diverse devices such as solar cells, photodetectors, and LEDs, among others.^4−6^ As the main driver of this evolution, lead-based perovskite solar cells (Pb-PSCs) have shown a rapid increment in power conversion efficiency (PCE), growing from 3.8%^2^ to 26.1%^7^ in only 14 years. However, the toxicity of Pb stands as a limiting drawback to the technological viability of many potential applications.^8^ Thus, in recent years, the research community has been investigating new options for replacing the Pb.

One of the most successful approaches involves substituting Pb^2+^ with Sn^2+^. Since both elements share the same valence electronic configuration (ns^2^np^2^), the resulting perovskites can also show comparable properties such as high charge carrier mobility, excellent light absorption, and a small exciton binding energy. However, perovskites containing Sn tend to have lower stability compared with their lead-based counterparts. Unlike Pb, which is commonly found as Pb^2+^, Sn can adopt multiple oxidation states, forming Sn^2+^ and Sn^4+^. This difference arises from the lanthanide contraction effect in Pb, caused by the presence of 4f^14^ electrons in its electronic configuration. In contrast, Sn lacks lanthanide contraction, making it easier for all valence electrons to be lost, thus resulting in the formation of Sn^4+^. As a result, Sn-PSCs present a very important challenge compared to their Pb-PSCs counterparts because of their facile oxidation of Sn^2+^ to Sn^4+^ under ambient conditions. That process produces Sn^2+^ vacancies in the perovskite structure and, hence, a high p-doping and high density of charge trap states,^9−13^ which dramatically damages the Sn-perovskite properties and limits the Sn-based perovskite stability. The use of reducing agents in the precursor solution has been investigated to avoid Sn^2+^ oxidation and improve the crystallinity of the perovskite films, such as hypophosphorous acid,^14^ gallic acid,^15^ tin halides (SnCl_2_ or SnF_2_),^16−18^ tin powder,^19^ phenylhydrazine hydrochloride,^20−22^ ascorbic acid,^23^ or sodium hydroborate.^9^ These proved to be an effective strategy to prevent the oxidation of Sn^2+^ to Sn^4+^.

Bulky ammonium cations, commonly used to form 2D perovskites,^24,25^ can also be incorporated into the perovskite solution to form more stable Sn-based perovskite thin films.^26,27^ These additives help in the crystallization process and protect the films due to the hydrophobic properties of the 2D cations, resulting in homogeneous and pinhole-free films.^28^ Several ammonium cations have been reported in the literature, such as ethylenediammonium (EDA),^29,30^ ethylammonium (EA),^31,32^ phenylethylammonium (PEA),^33,34^ 4-fluorophenethylammonium (FPEA),^35^ butylammonium (BA)^36,37^ or en-2-ethylammonium (TEA).^38−40^ In particular, the additive TEAX, where X is I, Br, or Cl, presents an additional defect passivation effect in Pb-based perovskites.^40−43^ TEAI was used in Sn-based perovskites to obtain 2D or 2D/3D perovskites^38,39,44−47^ and passivate bulk and surface defects. Although the incorporation of 2D cations is also a good strategy to control the crystallization process, the resulting thin films are prone to form a mixture of 3D/2D perovskites with a wider bandgap and worse charge extraction, making them less ideal candidates for solar cells.

Here, we implement the use of additive TEAX salts in FASnI_3_ solar cells, avoiding the formation of lower-dimensional phases, to combine stabilization of the crystal lattice with their defect passivation effect. These synergetic effects help stabilize the Sn^2+^, enhancing the device’s efficiency and improving stability under continuous operation.

Conventional FASnI_3_ perovskite presents a close-to-optimal bandgap for photovoltaic generation (1.4 eV) and can display excellent optoelectrical properties upon synthetic optimization. However, this material suffers from common Sn-based perovskite issues such as the fast and difficult-to-control crystallization dynamics that hinder the film quality or the oxidation of Sn^2+^ to Sn^4+^. Together, these factors compromise the long-term stability and efficiency of materials and related devices. The use of TEAI has been reported to improve the perovskite crystallization in Sn-PSCs, resulting in highly oriented 2D/3D tin-based perovskites with the addition of TEAI as a precursor^38,48^ or by the deposition of a bilayer.^40^ In this aspect, TEABr and TEACl additives remain less explored for Sn-based perovskites.^38−40^ Although layered lower-dimensionality TEAX-based Sn perovskites can show improved crystallization and enhance the ambient stability of the materials, they usually display a wider bandgap and worse charge transfer capabilities than their 3D counterparts, limiting the potential impact on photovoltaics.^25,49^ However, reducing the TEAX salt concentration in the solution can circumvent the formation of lower-dimensionality regions while maintaining the improved crystallization dynamics. In this aspect, amounts as low as 10 mol% of TEAX can be added into the perovskite solutions to control the crystallization of the FASnI_3_ films. Figure S1 shows the XRD patterns of TEA_*x*_FA_1–*x*_SnI_3_ perovskite for different mol% TEAI (100, 50, 20, 10, 1, and 0 mol%). With the addition of small TEAI concentrations (ca. 10 mol%), the resulting perovskite structure is represented by the peaks of pure 3D FASnI_3_. For higher TEAI concentrations (>10 mol%), the resulting TEAI-FASnI_3_ perovskite is a mixture of lower-dimensionality layered compounds. A combination of quasi-2D *n* = 1 and *n* = 2 peaks is observed, as well as those corresponding to the 3D phase. Finally, the pure 2D is found in the case of 100 mol% TEAI.

Hereafter, we will evaluate the samples containing the optimized concentration of TEAX, maintaining the 3D structure of the Sn-perovskite. These samples will be named based on the halide composition of the salt used as an additive, i.e., TEAI-, TEABr-, or TEACl-based ones. We will compare them to the reference FASnI_3_ sample without TEAX, referred to as the control.

Figure S2a–d displays top views of the films obtained using HRFESEM. All the films are compact. In particular, the TEAI-, TEABr-, and TEACl-based samples show smoother surfaces than the control FASnI_3_ film. The enhanced smoothness observed in TEAX-based perovskite films has the potential to diminish grain boundary effects and reduce carrier recombination, thereby enhancing ambient stability and charge transport in the solar cell, as reported elsewhere when forming the 2D/3D mixed structures.^50,51^

XRD diffractograms of the TEAX-based samples are displayed in Figure 1a. The characteristic peaks for pure 3D FASnI_3_ in the orthorhombic phase are observed in all cases, around 14.0° (100) and 28.0° (200).^52^ The XRD patterns show a significant increase in the intensity of these peaks for the samples with TEAX, indicating an improvement in the film crystallinity. A deeper analysis of the peaks reveals a reduction in the peak full width at half-maximum (fwhm) for the TEAX-based samples compared to the control one (Table S1), which confirms their improved crystallization with a reduction of the lattice strain dispersion.^53^ XRD peaks corresponding to the (100) and (200) planes in TEAI- and TEACl-based samples remain at the same position as those for the reference FASnI_3_ films for measurement resolution. In contrast, XRD patterns for the TEABr-based samples display a shift of the (100) plane to higher angles (zoomed-in gray zone in Figure 1a). The shift suggests that a small part of the Br^–^ ions are introduced into the perovskite lattice,^54,55^ while the I^–^ and Cl^–^ ions do not change the crystal lattice parameters. Energy-dispersive X-ray spectroscopy (EDS) analysis of the TEABr-based samples indicates that the S and Br atoms (the main distinguished elements of the salt) are distributed throughout the entire film, as represented in Figure S5.

**Figure 1:**
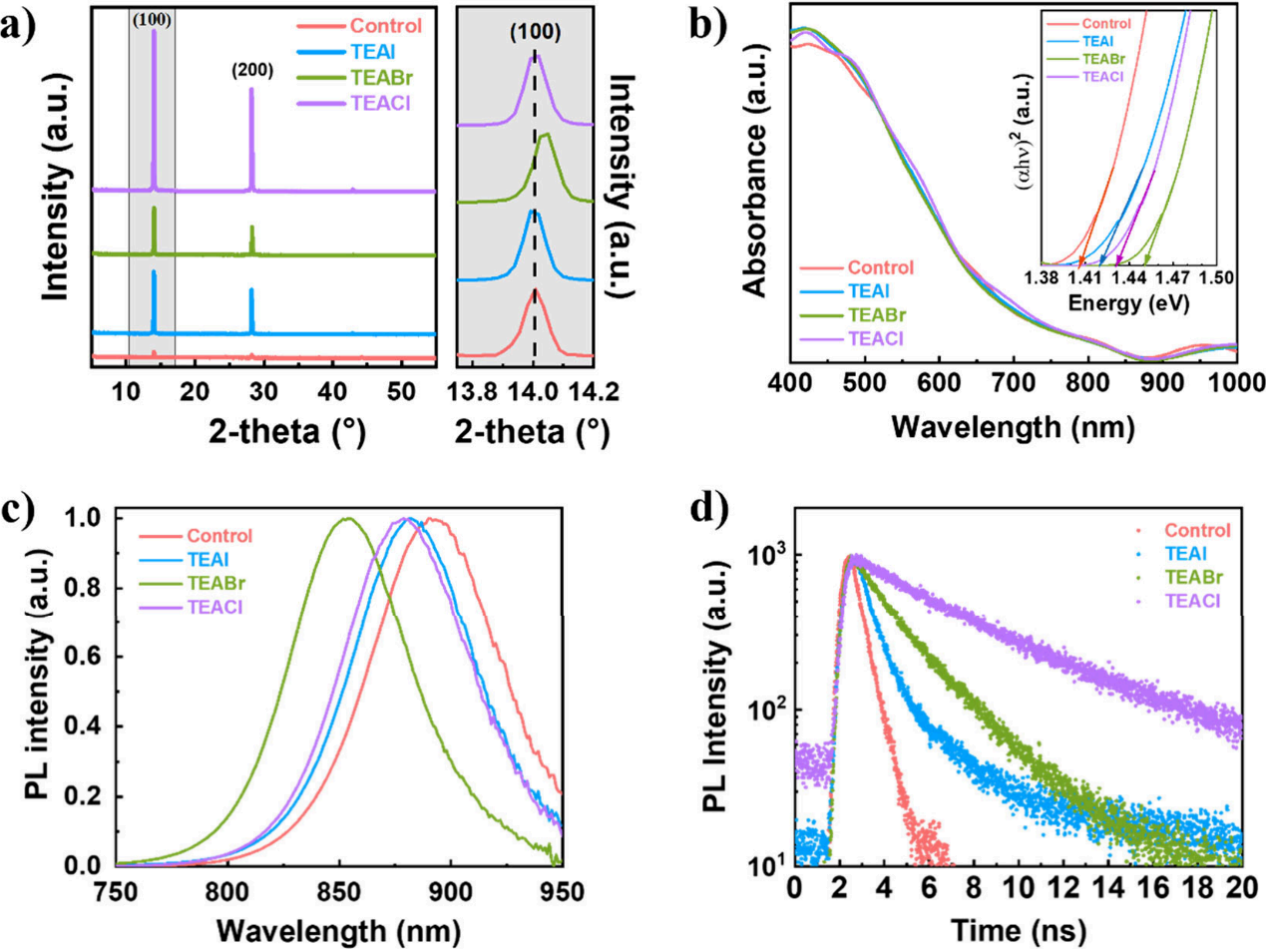
Perovskite film characterization of control, TEAI, TEABr, and TEACl: a) XRD patterns, b) UV–vis–NIR spectra, c) normalized photoluminescence, and d) time-resolved photoluminescence (in the case of the control sample, lifetime was calculated considering the instrument response function that causes the increase in PL intensity observed 5–8 ns after excitation).

Although the absorbance spectra of all the samples are similar (Figure 1b), the blue shift of the TEABr-based sample also indicates Br^–^ incorporation in the perovskite lattice. Nevertheless, the incorporation content is low, and only a slight blue-shift of the band edge absorption is observed, on the order of 50 meV with respect to the control sample, as displayed in the inset of Figure 1b. In fact, the optical absorption band edges obtained by the Tauc plots (inset of Figure 1b) are roughly around 1.40, 1.42, 1.43, and 1.45 eV for the control, TEAI-, TEACl-, and TEABr-based perovskite films, respectively. Such energy shifts are confirmed as well by steady-state photoluminescence (PL) spectra (normalized spectra in Figure 1c). While in the case of the TEABr-based sample this PL shift can be mostly due to the introduction of Br^–^ ions into the FASnI_3_ perovskite structure, for TEACl and TEAI the slight bandgap readjustment can be attributed to the above-mentioned trend in the dispersion of lattice strain.

The PL decay kinetics in individual films give us information about the photogenerated charge carrier dynamics (Figure 1d, measured at the maximum PL spectra). Single-exponential and biexponential fittings show the dominance of the short-lived components in all cases, which become the focus of the comparison. The PL lifetime (see Table S2) increases from 0.66 ns for the control sample to 0.99 and 1.71 ns for the TEAI- and TEABr-based perovskites, respectively, while a monoexponential fitting can be applied in the TEACl-based sample. This increase in the PL decay time is accompanied by a PL intensity enhancement (Figure S3), suggesting the reduction of nonradiative carrier recombination processes in the material.

Considering these potential performance improvements, TEAX-based films were tested as the active material for PV solar cells using a p-i-n configuration (ITO/PEDOT:PSS/Sn-PVK/C60/BCP/Ag). The cross-section images in Figure S4 display devices based on compact and pinhole-free films. The deposition conditions were adjusted (see Experimental Section in the Supporting Information) to obtain an optimal perovskite thickness around 200 nm, as previously reported.^56^Figure 2a displays the current–voltage (*J–V*) curves for the best solar cells, with all the perovskites measured in forward scan (dashed line) and reverse scan (solid line) under dark and 1 sun (100 mW/cm^2^ AM1.5G) illumination conditions. TEAX-based devices show improvements in all of the photovoltaic parameters in comparison with those of the control (FASnI_3_) solar cells. The best PCE was obtained with the TEABr-based device, displaying a PCE of 9.4%, i.e., 42% higher than that of the best control device. The most important change in the photovoltaic parameters occurs for *V*_oc_ . Analyzing the best photovoltaic samples in each study, the control device presents a *V*_oc_ of 0.42 V, whereas the rest of the additives show a slight enhancement for TEACl-based device (0.43 V), a significant improvement for TEAI-based device (0.50 V), and the most substantial enhancement for TEABr-based device, reaching 0.56 V. The fill factor (FF) also improves with the addition of TEAX, yet marginally, obtaining 70%, 72%, 73%, and 71% for the control, TEAI-, TEABr-, and TEACl-based solar cells, respectively. The *J*_sc_ for the best control device is around 21.6 mA/cm^2^, in contrast with the *J*_sc_ values for all of the best TEAX-based samples, which are higher than 23 mA/cm^2^. The external quantum efficiency (EQE) (Figure 2b) evidences an excellent photoresponse from the near-infrared to the UV region of the light spectra. This is in good agreement with the results obtained from the UV–vis–NIR absorbance spectra (Figure 1b). Similarly, the samples show good agreement between the *J*_sc_ obtained in the *J–V* curves and that obtained by the integrated EQE.

**Figure 2:**
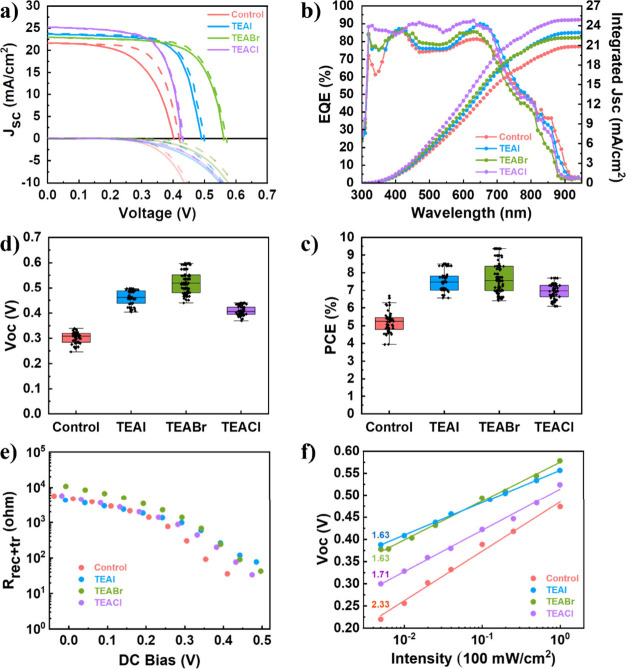
a) *J–V* curves under 1 sun and dark conditions for the best solar cells, b) EQE and integrated *J*_sc_, c) *V*_oc_ and d) PCE statistics with C_60_ as electron-selective contact, e) *R*_rec_ of the solar cells with C_60_ and equivalent circuit used to fit (as inset), and f) *V*_oc_ vs light intensity for solar cells with C_60_ as electron-selective contact.

In order to confirm these observations and minimize potential errors derived from device-to-device variability, the photovoltaic performance was statistically analyzed through the fabrication of ∼50 devices for each additive. The *V*_oc_ and PCE statistics are shown in Figure 2c,d, and the *J*_sc_ and FF statistics are displayed in Figure S6a,b. All of the photovoltaic parameters improve upon TEAX addition, but the most important enhancement was observed in the *V*_oc_. While the FF and *J*_sc_ improvements can be due to improved charge extraction mechanisms due to the better crystallization and surface morphology in the perovskite films, as suggested by the SEM images (Figure S2) and XRD diffractograms (Figure 1a), the changes of *V*_oc_ can be attributed to recombination processes. Low *V*_oc_ is a usual issue in Sn-based PSCs, often resulting from the trap densities generated by the oxidation of Sn^2+^ to Sn^4+^. This oxidation process may produce charge recombination centers within the bulk and/or on the perovskite surfaces, resulting in an increase of nonradiative recombination.^13,57−59^

To further understand the charge dynamics that induce voltage improvements in the devices, impedance spectroscopy (IS) was measured at different DC bias potentials upon exposure to 1 sun in ambient conditions. The resulting Nyquist plots (see Figure S7) are concordant with the characteristic patterns expected for Sn-PSCs. However, it is important to remark that some samples present nonconventional behaviors close to *V*_oc_ at low frequencies (Figure S7b), the study of which is out of the scope of this work. The spectra were fitted using an equivalent circuit reported elsewhere^60^ that includes a series resistance (*R*_s_) that is related to the resistances of the electrodes and wires, a resistor (*R*_rec_) that couples both the recombination and the transport resistances but is dominated by the recombination process in samples with optimal transport such as the ones analyzed here, the geometric capacitance (*C*_g_) as a classical capacitor with electrostatic nature of the films, and a branch with a capacitor (*C*_ion_) and resistor (*R*_ion_), both related to the ionic nature characteristic of the halide perovskites. The *R*_rec_ and the circuit used for the fitting are shown in Figure 2e. The addition of TEAX increases the *R*_rec_ in all cases. This increment in resistance indicates a decrease in the charge recombination rate, which pinpoints the origin of the *V*_oc_ improvement. Thus, the lower *R*_rec_ of the control device indicates a larger recombination rate, in agreement with the photoemission analysis of the perovskite films that show higher nonradiative recombination rate in the control sample (see Figure S3). The *V*_oc_ dependence with the logarithm of the light intensity (Figure 2f) offers further details on the recombination mechanism. The deviation from *k*_B_*T*/*e* (where *k*_B_ is the Boltzmann constant, *T* is the temperature, and *e* is the elementary charge) of the slope is measured by the ideality factor. Values closer to 1 imply no slope deviation from *k*_B_*T*/*e*, representing a direct recombination, while deviations with values closer to 2 are usually attributed to trap-assisted recombination.^61^ In Figure 2f, TEAX-based solar cells present ideality factors of 1.63, 1.63, and 1.71 for TEAI, TEABr, and TEACl, respectively. These values are smaller than the one in the control solar cell (2.33), which can be interpreted as a reduction of the bulk trap-assisted recombination as a result of the reduction of bulk recombination centers.^61,62^

This point must be corroborated by compositional analysis, such as X-ray photoelectron spectroscopy (XPS). This is an excellent method for evaluating the surface of the perovskite film and assess the ambient stability of the Sn-perovskite films, which is one of the main issues in these systems. Figure S8 displays the XPS spectra for S 2p, Br 3d, and Cl 2p and confirms the presence of these elements in the perovskite films. With the focus on the key component, Figure 3a,b shows the Sn 3d XPS spectra before and after exposure to ambient conditions (∼65% RH and 30 °C) for 120 min. In the Sn 3d XPS spectra of the control sample, two peaks at 486.5 and 487.7 eV are deconvoluted from the Sn 3d^5/2^ peaks. These peaks are identified as corresponding to Sn^2+^ and Sn^4+^, respectively. Similar peaks can be observed from the TEAX-treated films. Before ambient exposure, fresh perovskites display a relatively low Sn^4+^ content. While the Sn^2+^/Sn^4+^ ratio of the control samples is 92%, the Sn^2+^/Sn^4+^ ratio increases to 97% upon the addition of TEAX, confirming a lower level of Sn^4+^ formation. More interestingly, the control FASnI_3_ film undergoes complete degradation after exposure to ambient conditions for 120 min (Figure 3b), revealing a Sn^2+^/Sn^4+^ ratio of 6%. In contrast, the TEAI-, TEABr-, and TEACl-based samples exhibit significantly higher Sn^2+^/Sn^4+^ ratios of 80%, 87%, and 81%, respectively, an indication of better resistance to ambient-induced degradation. In Figure 3c, the Sn^2+^/Sn^4+^ ratio is depicted for various exposure times (see XPS in Figure S9), providing insights into how Sn^2+^ oxidation evolves over time under ambient conditions. Clearly, the addition of TEAX additives prevents the oxidation of Sn^2+^ to Sn^4+^, significantly enhancing the FASnI_3_ stability. This effect is particularly pronounced when TEABr is used. This enhanced stability is of paramount importance in the development of efficient and long-lasting perovskite solar cells.

**Figure 3:**
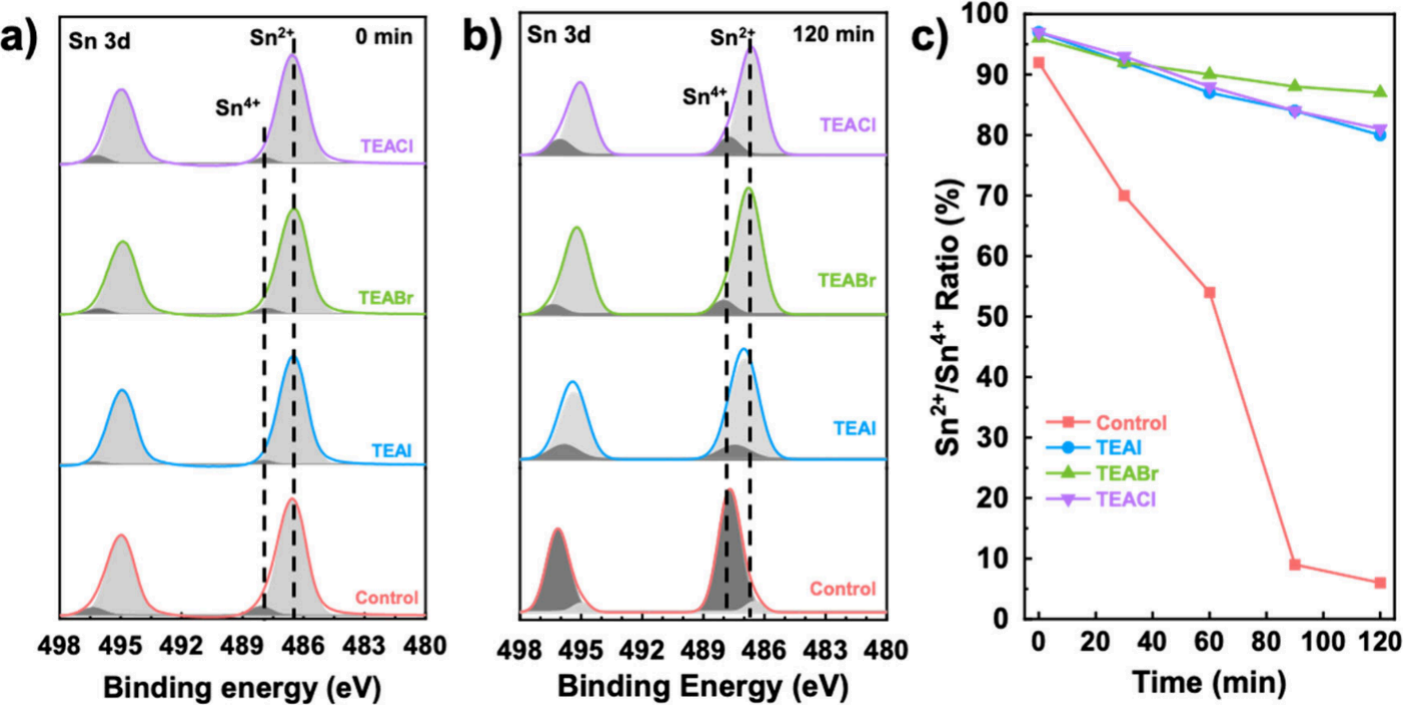
a) Sn 3d XPS spectra for the fresh perovskite films, b) Sn^2+^ content evolution of the perovskite films exposed to ambient conditions at different times, and c) Sn^2+^/Sn^4+^ ratio evolution as a function of exposure time in ambient conditions.

The enhanced crystallinity and stability of Sn^2+^ in TEAX-treated FASnI_3_ may be attributed to interactions between thiophene and Sn^2+^. Nuclear magnetic resonance (NMR) measurements of the precursor solutions (Figure S10) show an upshift from the −711 ppm peak (for control) to −765 ppm (when TEABr is added) that suggests the formation of a complex due to the electrons donated from the sulfur to the tin.^44,63,64^ Thiophene, as present in TEAX, is an electron-rich heterocycle compound containing a sulfur atom in its ring structure. Generally, S-containing compounds can serve as ligands, donating their lone pairs of electrons to form strong coordination bonds with metal ions like Sn^2+^. Thus, the improvement in film crystallinity is linked to the adjustment of the crystallization dynamics upon the formation of this complex, similar to the effect observed with DMSO adduct formation.^28^ Yet as an additional effect, the thiophene can effectively shield Sn^2+^ from further oxidation to Sn^4+^ by solvents like DMSO and oxidizing atmosphere, thus preserving the stability of the FASnI_3_.^40,42,43,48,65^

Therefore, it is crucial to test whether the enhanced resistance to Sn^2+^ oxidation leads to an improved stability of the solar cells’ performance. The evolution of the control and TEABr-based solar cells’ performance measured under continuous operation (1 sun AM1.5G illumination and alternating continuous maximum power point conditions and *J–V* curves) is shown in Figure 4a. The measurements are performed under a N_2_ atmosphere to simulate an optimal encapsulation of the devices. The control device’s performance oscillates slightly during the initial 100 h of continuous operation and decays after 1000 h, reaching a T80’ (time to achieve the 80% of the initial efficiency) of ∼1250 h. In contrast, the TEABr-based solar cell (a device with average parameters, purposefully chosen to enhance the generality of the analysis, initial PCE = 7.18%) shows a significant enhancement of the PV performance during the first 50 h, followed by a stabilization at a gain of 10% of the initial value. This phenomenon can be attributed to the light soaking process, as previously reported.^9^ The efficiency plateau is maintained for >2000 h at a performance larger than the initial one, with a sudden drop resulting in a T80’ of ∼2300 h, one of the highest values obtained for Sn-based PSCs under 1 sun AM1.5G continuous operation.

**Figure 4:**
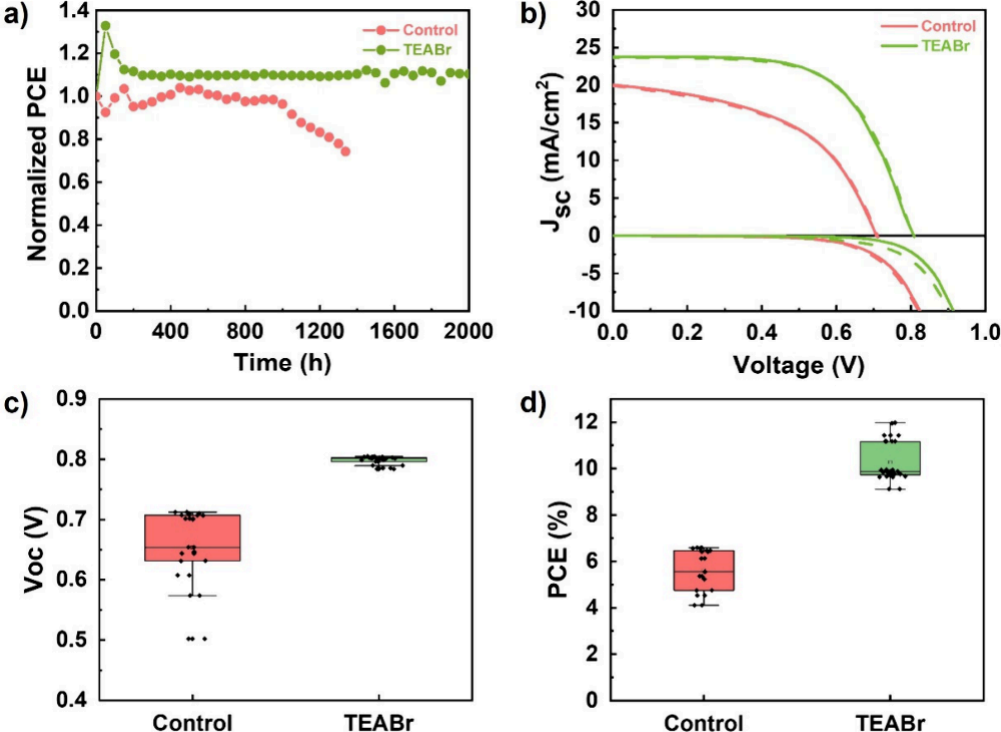
a) Stability of the control and TEABr-based solar cells under N_2_ conditions at room temperature, b) *J–V* curves under 1 sun and dark conditions for the best solar cells in forward scan (dash line) and reverse scan (solid line), and c) *V*_oc_ and d) PCE statistics with ICBA as electron-selective contact.

The best-performing device, based on the TEABr additive, is also the device with the highest *V*_oc_, in line with the hindered Sn^4+^ formation. However, additional losses related to a suboptimal band energy alignment using C_60_ as electron-selective contact^66−69^ can limit the *V*_oc_. The use of ICBA as an electron-selective contact presents a better band energy alignment, which can be used as leverage to enhance the TEABr-based device *V*_oc_, one of the Achilles heels of Sn-based PSCs. Figure 4b shows the *J–V* curves for the best devices under dark and 1 sun conditions when C_60_ is substituted by ICBA. The *V*_oc_ and PCE statistics (∼50 devices) are shown in Figure 4c and d, and the FF and *J*_sc_ statistics are shown in Figure S11a and b, respectively. As clearly displayed in Figure 4c, the TEABr-based devices show a significant improvement in the *V*_oc_ with high reproducibility in comparison with the control devices with the same electron-selective contact. This enhancement is reflected in the dependency of this parameter with the illumination intensity; see Figure S11c. Interestingly, the improvement of the interface with the ICBA shifts the ideality factor of these samples back to ∼2, in contrast to the smaller ideality factor in the TEAX sample with C_60_ (Figure 2f). This behavior reflects the improvement of the interface, which is no longer the limiting factor, shifting the main recombination path to bulk traps that can be related to the low conductivity of the ICBA due to the disorder of the isomers.^70^ In addition, the improved configuration with ICBA maximizes the differences between the TEABr-based (well passivated, less Sn^4+^ content) and control samples, as expected when both devices are dominated by trap-mediated bulk recombination. The champion TEABr-based cell with this configuration shows higher *J*_sc_ than the control FASnI_3_ sample (23 and 20 mA/cm^2^ for the TEABr-based and control devices, respectively). Figure S11d shows the EQE and the integrated *J*_sc_, which matches the one measured in the *J–V* curves like in the case of samples with C_60_ as electron-selective contact. However, a small reduction of FF, probably related to a hindered charge extraction mechanism with the ICBA, limits overall PCE improvement.

As a combination of these parameters, the best PCE obtained was 6.8% and 12.0% for the control and TEABr-based devices, respectively, with a significant *V*_oc_ enhancement (0.8 V for the TEABr-based perovskite and 0.7 V for the control FASnI_3_). The PCE in these solar cells shows an increment, obtaining an average PCE of 5.6 ± 0.9% and 10.3 ± 0.8% for the control and TEABr-based solar cells, respectively (see Figure 4d). The average of all parameters is shown in Tables S3 and S4 for all perovskite solar cell conditions with the different electron-selective contacts, where the improvement of all figures of merit upon TEAX addition is evident.

The addition of TEAX into the perovskite precursor solutions results in a better control of the films’ crystallization, obtaining a 3D phase of FASnI_3_ when the optimal TEAX concentrations are used. The incorporation of these additives also generates a significant Sn^4+^ content reduction in the fresh samples. These effects are attributed to the S–Sn interaction in the solution that can prevent the Sn^2+^ oxidation. As a result, TEABr-based solar cells keep >95% of the initial efficiency for more than 2000 h under continuous operation at 1 sun illumination and N_2_ environment. The maximum PCE obtained when C_60_ is used as the electron-selective contact was 6.6% with the control perovskite and 9.4% (>40% enhancement) with the addition of TEABr. With the interfacial improvement resulting from the substitution of C_60_ by ICBA, a maximum efficiency as high as 12% was reached with TEABr-based samples. Although all of the parameters are clearly improved upon the addition of TEABr, the most important change was found for *V*_oc_. Its origin is found in the reduction of the Sn^4+^ content and concurrent elimination of nonradiative recombination channels, as proved by the IS results and by the increment in the PL intensity and longer carrier recombination times. This work offers an easy way to control the crystallization and reduce the defect density in lead-free perovskites. Our approach sets a novel route to improve Sn-based PSC efficiency and stability with the introduction of S-based long-chain organic cations for passivation plus stabilization of Sn^2+^.

## Supporting Information Available

Experimental details and additional characterization results: XRD, HRFESEM, PL, EDS, statistics, Nyquist plots, XPS, NMR, ideality factor, and EQE (PDF)
