# Supplementary material for: Adjusting the Crystallization of Tin Perovskites through Thiophene Additives for Improved Photovoltaic Stability
Source: ACS Energy Lett. 2024 Oct 8;9(11):5288–95. doi: 10.1021/acsenergylett.4c01875 (PMC11558796; doi:10.1021/acsenergylett.4c01875)
Supplement: Supplementary file 1 — nz4c01875_si_001.pdf [file nz4c01875_si_001.pdf]

Supporting Information

Adjusting the crystallization of tin-perovskites through thiophene additives for improved photovoltaic stability

Omar E. Solis[1], Miriam Mínguez-Avellán[1], Pablo F. Betancur[1], Raúl I. Sánchez-Alarcón[1], Isabelle Rodriguez[2], Juan P. Martínez-Pastor[1], Teresa S. Ripolles[1*], Rafael Abargues[1*] and Pablo P. Boix[2*]

[1] Instituto de Ciencia de los Materiales de la Universidad de Valencia (ICMUV), 46980, Paterna, València, Spain.
[2] Instituto de Tecnología Química, Universitat Politècnica València-Consejo Superior de Investigaciones Científicas, Av. dels Tarongers, 46022, València, Spain.

Experimental section

Materials. Tin iodide (SnI_2, 99.999%), tin fluoride (SnF_2, 99%), N,N-dimethylformamide (DMF, ≥99.8%, anhydrous), dimethyl sulfoxide (DMSO, 99.9%, anhydrous), chlorobenzene (CB, 99.8%, anhydrous), hydroiodic acid (HI, 57%), hydrobromic acid (HBr, 48%), hydrochloric acid (HCl, 37%) and diethyl ether (DE, ≥99) were purchased from Sigma Aldrich. Formamidinium iodide (FAI, $HC(NH_2)_2I$, 99.99%) was purchased from Greatcell Solar Materials. 2- thiopheneethylamine (TEA, > 98%) was purchased from TCI Chemicals. Fullerene (C_{60}), di[1,4]methanonaphthaleno[1,2:2',3';56,60:2'',3''][5,6]fullerene-C_{60}-Ih, 1',1'',4',4''-tetrahydro- (ICBA) and bathocuproine (BCP) were purchased from 1-Materials. PEDOT:PSS AI 4083 aqueous dispersion was purchased from Heraeus. All the purchased chemicals were used as received without further purification.

Synthesis of TEAX (X= I, Br and Cl). Briefly, 20 mL of methanol and 1.2 mL of TEA (0.01 mol) were mixed in a 50 mL round bottom flask at room temperature under magnetic stirring. After that, reaction mixture was purged under a nitrogen flux and subsequently, 1 molar equivalent of HX (X= Cl, Br, I) were injected and stirred for 30

min. Finally, methanol was removed by evaporation in vacuum. TEAX powders were washed by filtration with DE three times and dry overnight at 60°C in vacuum.

Preparation of perovskite solutions. The control perovskite precursor solution was prepared by dissolving 0.4 mmol of SnI_2 (149 mg), 0.4 mmol of FAI (68.8 mg) and 0.04 mmol of SnF_2 (6.2 mg) in 1 mL of a mixed solvents of DMF:DMSO (4:1 in volume). The TEAX-based perovskite solution was prepared by dissolving 0.4 mmol of SnI_2 (149 mg), 0.36 mmol of FAI (61.9 mg), 0.04 mmol of SnF_2 (6.2 mg) and 0.08 mmol of TEAX (20.4, 16.6 and 13.1 mg for TEAI, TEABr and TEACl, respectively) in 1 mL of a mixed solvents of DMF:DMSO (4:1 in volume).

Device fabrication. Patterned ITO substrates (2.0 × 1.5 cm^2, 6-8 ohm/sq, BIOTAIN CRYSTAL CO., LIMITED) were washed in an ultrasonic bath for 15 min per process. First, the substrates were washed in water with soap and rinsed with distilled water. Then, the substrates were washed with ethanol, acetone, and isopropanol, sequentially. After drying under nitrogen flow, the substrates were treated for 30 min with UV-O_3. The hole transport material (HTM) PEDOT:PSS was filtered with 0.45 μm PVDF filter and spin coated on top of the ITO substrate at 5000 rpm for 30 s and annealed at 130 °C for 20 min in ambient conditions. After HTM deposition, the substrates were transferred into a N_2-filled glovebox for the perovskite deposition.

The perovskite solutions were filtered with 0.22 μm PTFE filter, the films were deposited using 30 μL of the precursor solution and spin coated with two steps, firstly, 1000 rpm for 10 s and, secondly, 5000 rpm for 50 s. After 22 s of having started the second step, 150 μL of CB was deposited like antisolvent and the annealing treatment was carried out at 100 ºC for 20 min. The electron transport material (ETM) C_{60} (30 nm) was thermal evaporated under a pressure less than 10^{-6} mbar. ICBA solution (20 mg/mL dissolved in CB) was deposited by spin coating on top of the perovskite film at 1400 rpm for 30 s and

annealing at 100 °C for 10 min. Finally, the samples were transferred into a vacuum chamber, BCP (6 nm) and Ag (100 nm) were sequentially deposited by thermal evaporation under a pressure less than 10^{-6} mbar.

Film and device characterization.

SEM images were taken with a High-Resolution Field Emission Scanning Electron Microscope (HRFESEM) ZEISS GeminiSEM 500 at 1 kV.

XRD pattern of the perovskite films were measured using X-ray diffractometer (PANalytical Empyrean X-ray) (Cu Kα, wavelength λ=1.5406 Å) with a Bragg angle range of 4-60° and step size of 0.026°.

Absorption spectra were measured with a JASCO V-770 spectrophotometer with the wavelength range from 300 nm to 1000 nm with a step size of 5 nm.

PL characterization was performed with an Edinburgh Instrument FLS 1000 PL spectrometer with double-grating Czerny-Turner monochromator for excitation and detection, the latter spectrally dispersing PL light into a high-speed PMT in a cooled housing for measuring both PL and Time Resolved PL (TRPL) spectra. In the latter case, the pulsed excitation at 470 nm was delivered by a fiber coupled super-continuum laser (FYLA SCTHP SN1121) attached to one of the two entry ports of the FLS 1000 excitation arm, with a pulse duration of about 10 ps, excitation fluence of about 1 nJ/cm^2 and pulse repetition rate of 40 MHz. For steady-state PL spectra the excitation was performed at 405 nm using a 400 W Xe lamp attached to the second entry port of the FLS 1000 excitation arm.

XPS measurements were performed in a Thermo Scientific K-alpha X-ray photoelectron spectrometer system (based pressure 4x10^{-9} mbar). Photoelectrons were excited with Al

K-α line 1486.6 eV of a monochromatized X-ray source. Measurements were taken at room temperature with a pass energy of 20 eV, with a spot size 400 μm.

J-V curves measurements in ambient conditions were taken with a Ossila solar simulator class AAA and automated J-V measurement system (T2003B3-G2009A1). The light intensity was adjusted to 1 sun (100 mW/cm^2) using a calibrated Si solar cell (RERA solutions, RQN3290, RQN001). The solar cells were measured in ambient conditions (T~30°C and RH≥60%) without any encapsulation, with an active area of 0.084 cm^2 defined by a mask. The cell stability was measured under ambient and N_2 conditions. The stability procedure is described as follows. An initial J-V curve was measured to determine the maximum voltage (V_{max}) and this voltage was applied to the solar cell during 1 h at continuous illumination and after that hour another J-V curve was measured for determine, once again, the V_{max} and applied the new V_{max}. The parameters of the solar cell were monitored and registered every J-V curve.

The external quantum efficiency (EQE) was measured from the absolute spectral response (ASR). The ASR was measured using a Xe lamp solar simulator (Zolix 150W). An optical chopper (SCITEC INSTRUMENTS), monochromator (Omni-λ150), the Ossila test board (P2008A1) were used to connect the device. A Si calibrated photodiode (ThorLabs FDS100) was used as reference sample. ASR was measured with a lock-in amplifier (STANFORD RESEARCH SYSTEMS, SR810DSP). The monochromator and the lock-in were controlled through LabView software. EQE was calculated with the spectral response with the next equation,

$$EQE_{(\lambda)} = \frac{I_{sc_{sample}}}{I_{sc_{ref}}}(\frac{1240}{\lambda})(ASR)$$

Where, $EQE_{(\lambda)}$ is the external quantum efficiency for each wavelength. $I_{sc_{sample}}$ and $I_{sc_{ref}}$ are the currents generated for the sample and the reference sample, respectively. λ is the wavelength and ASR is the absolute spectral response.

For the integrated J_{sc} from the EQE, next equation was used:

$$J_{sc_{(\lambda)}} = \int \left[(EQE_{(\lambda)})(AM1.5G_{(\lambda)})(\frac{\lambda}{1240})\right] d\lambda$$

Where, $J_{sc_{(\lambda)}}$ is the short circuit current density, EQE is the external quantum efficiency, AM1.5G is the solar spectra irradiance, and λ is the wavelength. All parameters are in function of the λ.

Impedance spectroscopy was carried out using a Gamry 1010E potentiostat/galvanostat on complete solar cells at room conditions under 1 sun illumination. The measurements were performed at different offset voltages spaced 50 mV from the open circuit to 0 V, with a 10 mV AC perturbation ranging between 1 MHz to 0.01 Hz. Z-view software was employed to analyze the results and fit the data to the equivalent circuit.

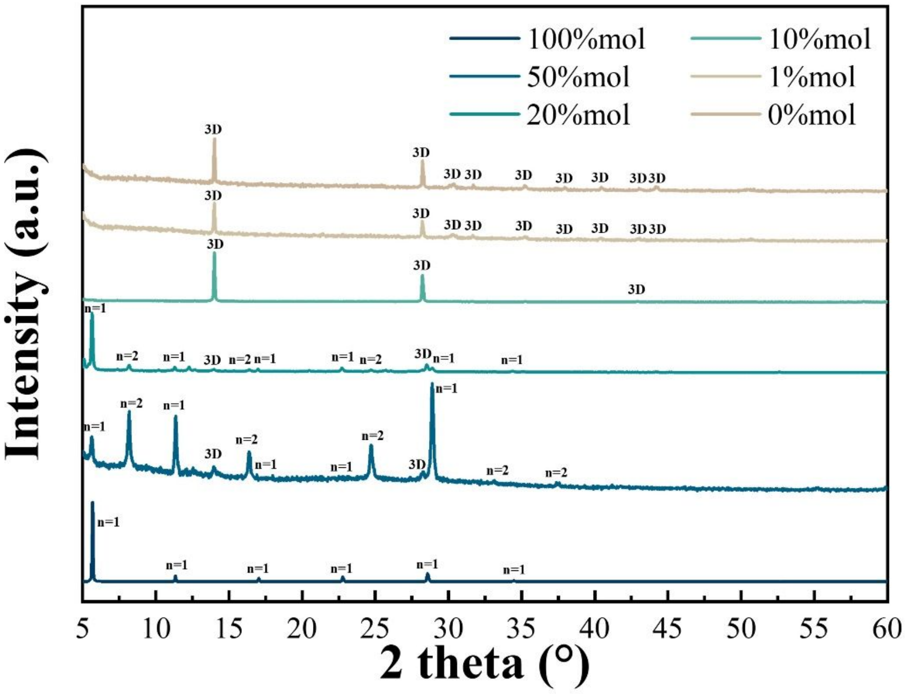

Figure S1.- XRD pattern for the $FASnI_3$ with different concentrations of TEAI.

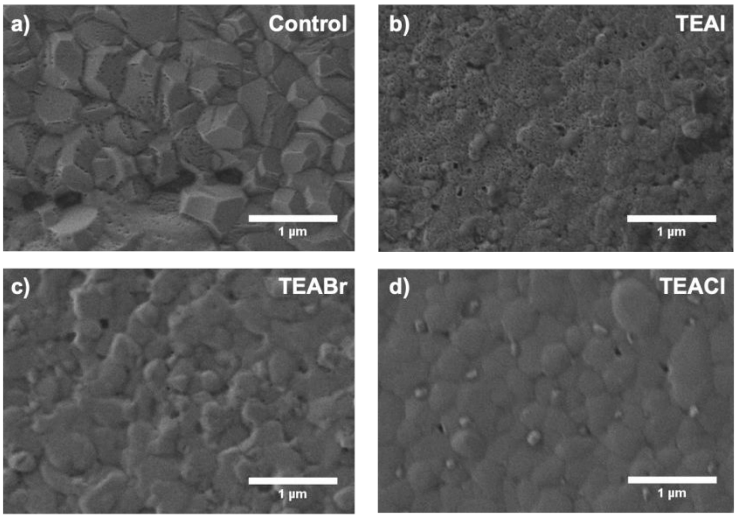

Figure S2.- Perovskite film characterization. a)-d) top view SEM images of control, TEAI, TEABr and TEACl-based samples, respectively.

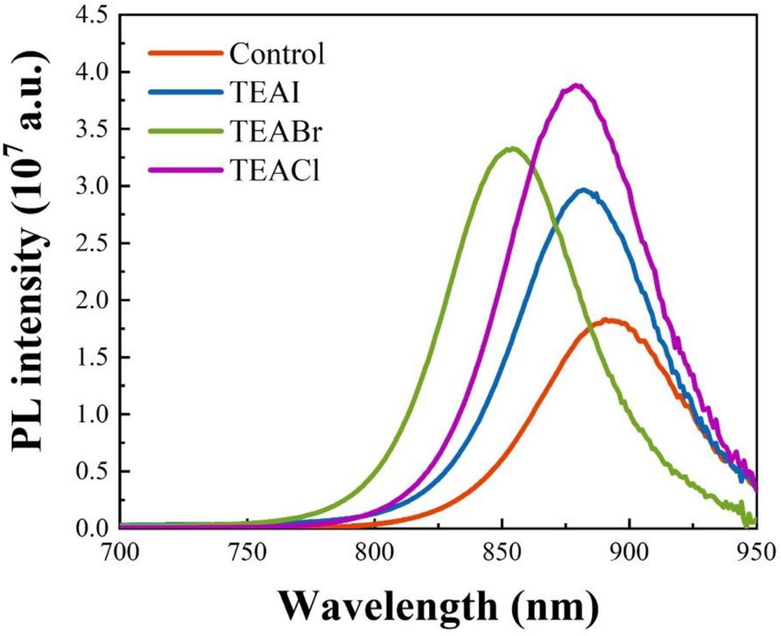

Figure S3.- PL intensity for control, TEAI-, TEABr- and TEACl-based perovskite films.

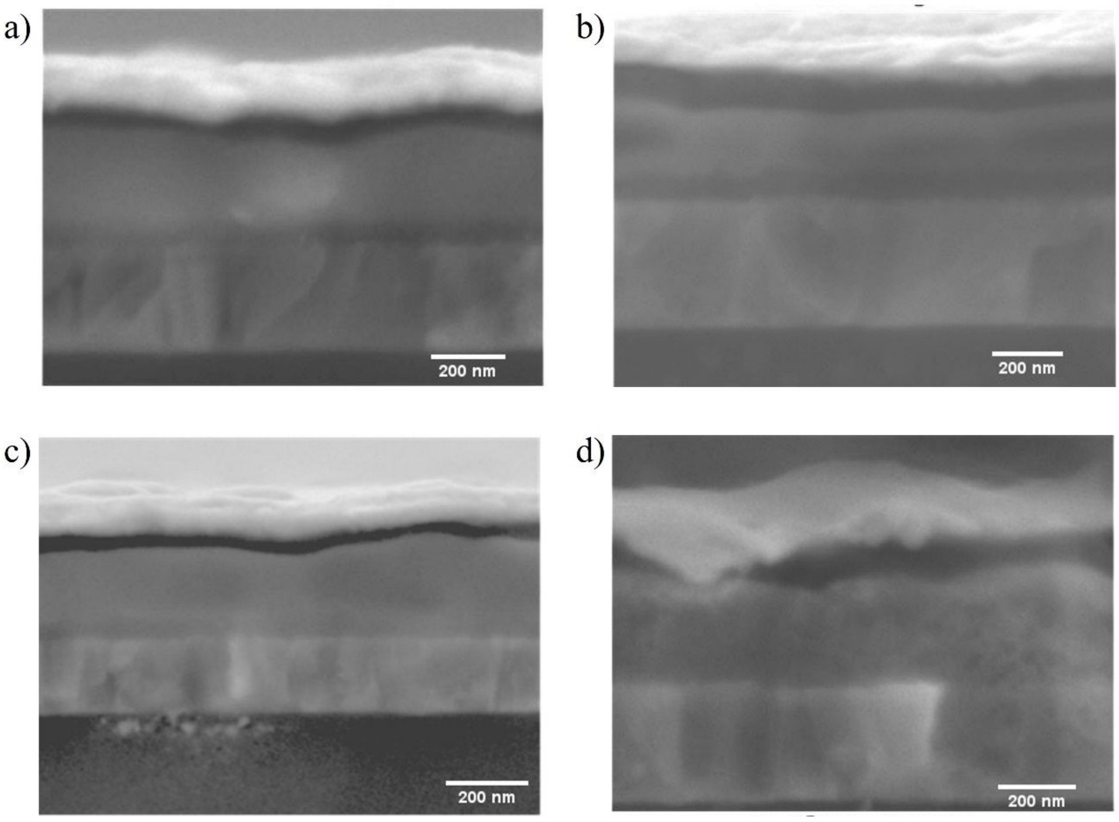

Figure S4.- HRFESEM Cross-section images of a) control, b) TEAI-, c) TEABr- and d) TEACl-based devices with C_{60} as ETL.

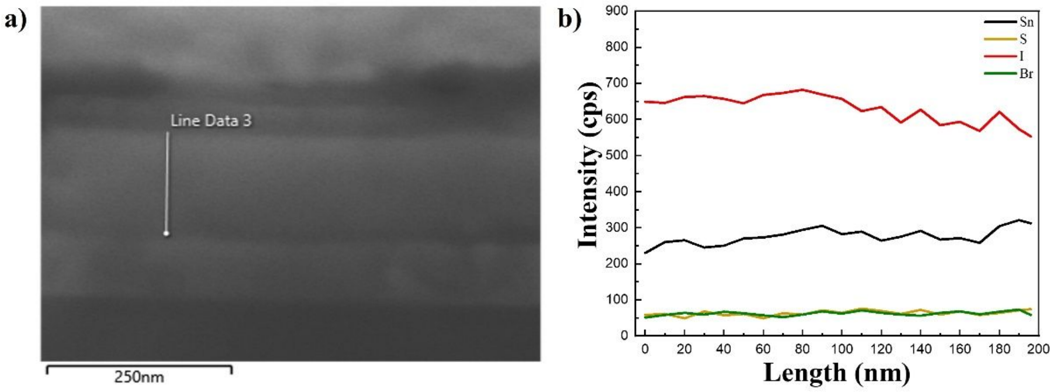

Fig S5.- a) Cross section image of TEABr-based device and b) EDS analysis along the perovskite film.

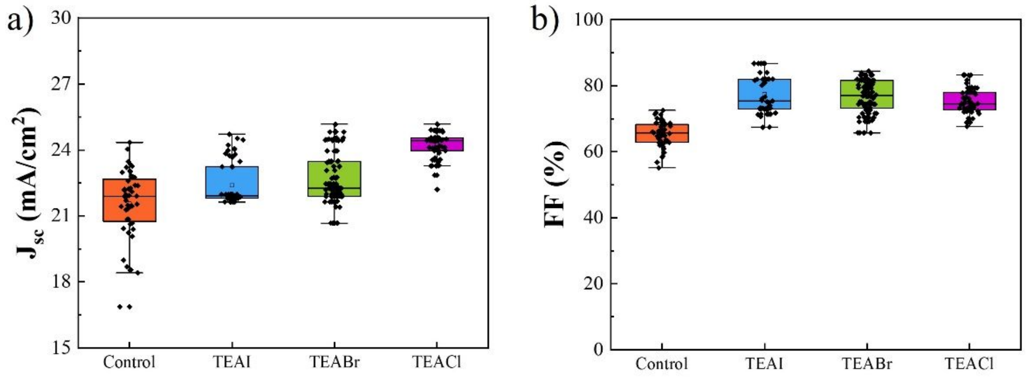

Figure S6.- Statistics of a) J_{sc} and b) FF for devices with C_{60} as ETL.

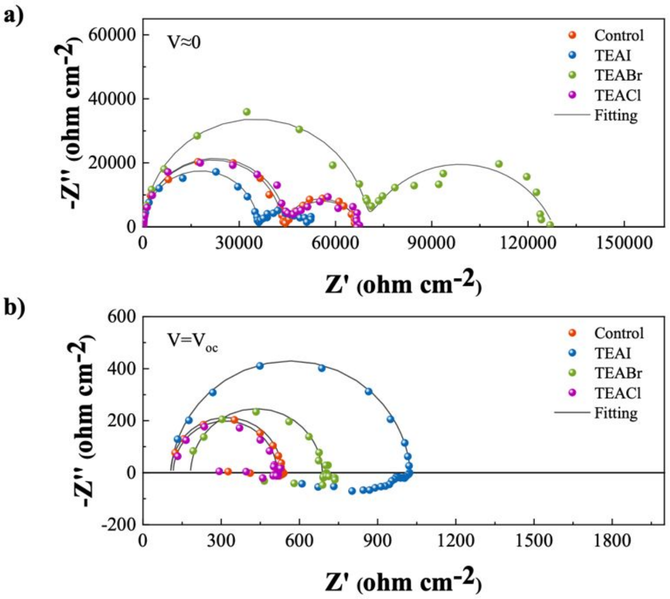

Figure S7.- Nyquist plots of the perovskite solar cells at a) V_{bias}= 0 V and b) V_{bias}=V_{oc} under 1 sun illumination in ambient conditions. The solid lines indicate the fitting using the circuit depicted in Figure 3c of the main text.

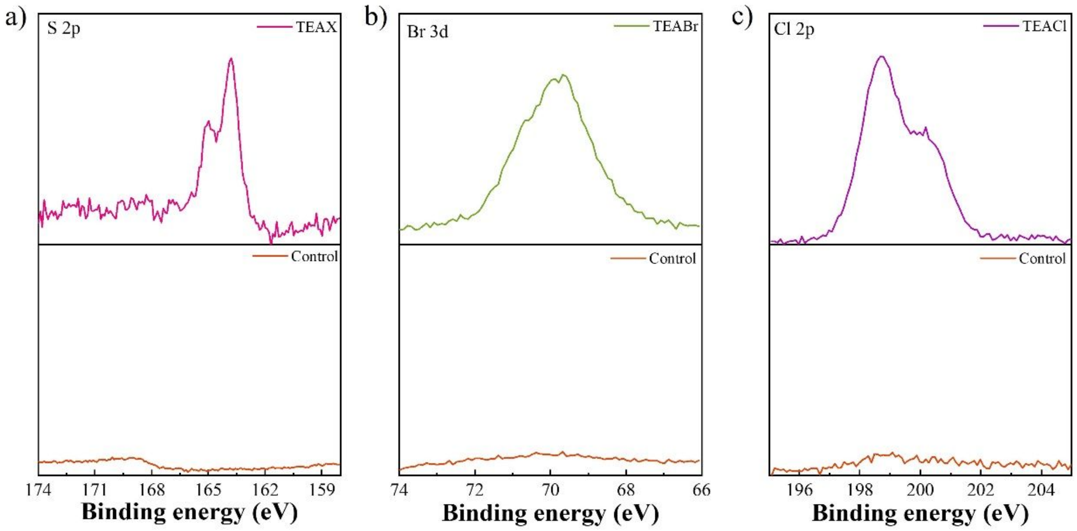

Figure S8.- XPS spectra of the TEAX-based perovskite films of a) S 2p and b) Br 3d from the TEABr and c) Cl 2p from the TEACl.

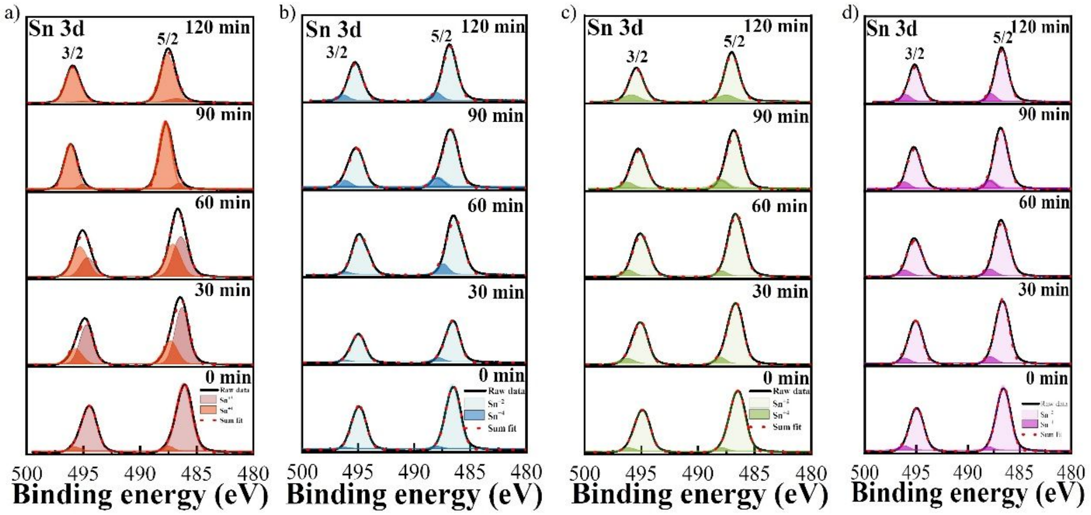

Figure S9.- XPS spectra evolution of the perovskite films a) control, b) TEAI-, c) TEABr- and d) TEACl-based at different times of exposure to ambient conditions.

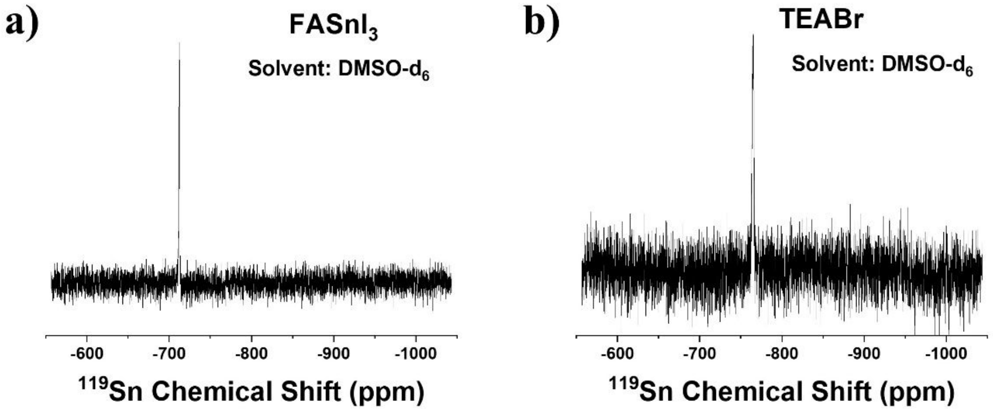

Figure S10.- NMR of ^{119}Sn measurements for a) control and b) TEABr-based perovskite solutions.

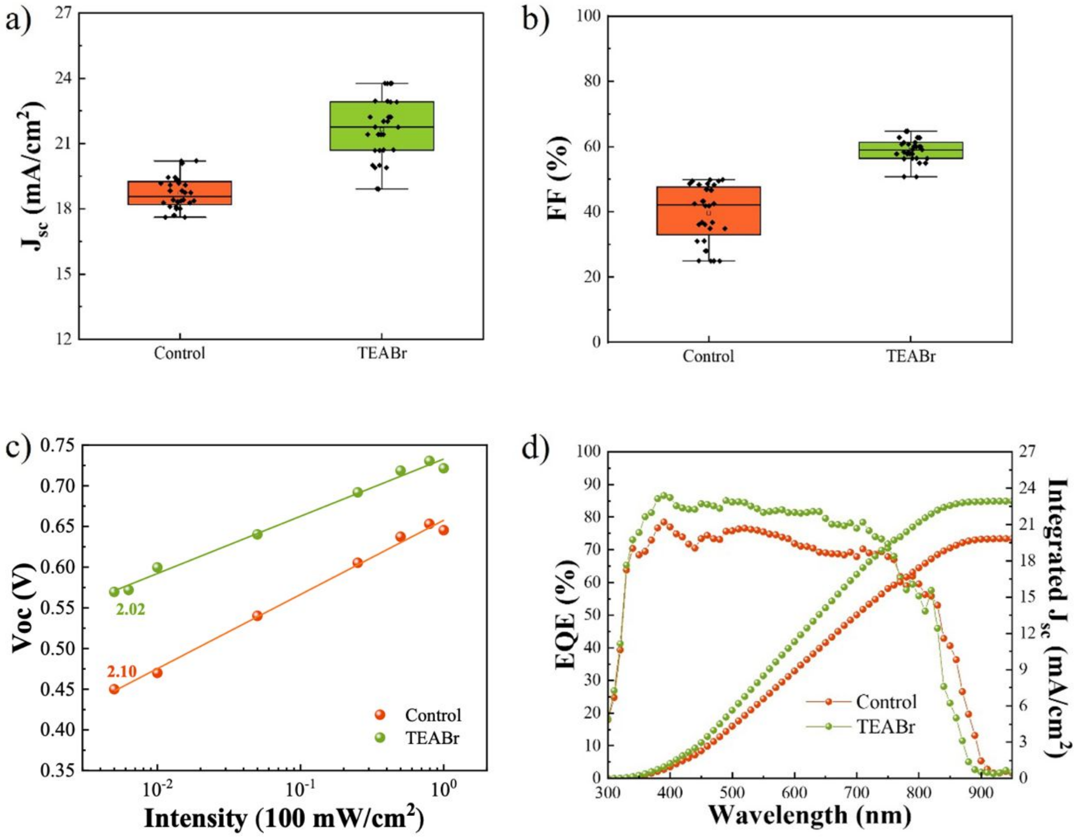

Figure S11.- Statistics of a) J_{sc}, and b) FF, c) Voc vs light intensity and d) EQE and integrated J_{sc} for devices with ICBA as ETL.

Table S1. XRD pattern indicating the strain-dispersion for different conditions.

Sample	2-theta	FWHM	Band Gap from PL
Control	14.011	0.09857	1.40
TEAI	14.001	0.08637	1.42
TEABr	14.034	0.08148	1.45
TEACl	14.008	0.08805	1.43

Table S2. Fitted PL lifetimes and their contributions to each term of the biexponential formula.

	τ_1 (A_1)	τ_2 (A_2)
Control	0.66 ns (100 %)	
TEAI	0.99 ns (99 %)	12.44 ns (1 %)
TEABr	1.71 ns (89 %)	4.63 ns (11 %)
TEACl	5.16 ns (100 %)	

PL decay kinetics were fitted to a biexponential decay as follows:

$$y(t) = A_1 e^{\frac{-t}{\tau_1}} + A_2 e^{\frac{-t}{\tau_2}} + y_0$$

The monoexponential fitting is obtained by eliminating contribution 2 from previous formula. In the case of the control sample, lifetime was calculated considering the instrument response function (IRF) that causes the increase in PL intensity observed 5-8 ns after excitation (see Figure 2d in main text).

Table S3.- Average of the parameters of the solar cells with C_{60} as ETL.

Sample	V_{oc} (V)	J_{sc} (mA/cm^2)	FF (%)	PCE (%)	Best PCE (%)
control	0.4±0.1	21.5±1.5	56±3	5.2±0.6	6.7
TEAI	0.5±0.1	22.4±0.9	70±5	7.5±0.6	8.5
TEABr	0.6±0.1	22.6±1.1	70±4	8.5±0.4	9.4
TEACl	0.4±0.5	24.3±0.6	68±3	6.9±0.4	7.7

Table S4.- Average of the parameters of the solar cells with ICBA as ETL.

Sample	V_{oc} (V)	J_{sc} (mA/cm^2)	FF (%)	PCE (%)	Best PCE (%)
control	0.6±0.1	18.7±0.8	40±9	5.6±0.9	6.6
TEABr	0.8±0.01	21.6±1.4	58±3	10.3±0.8	12

Table S5.- Parameters of the control perovskite solar cells with C_{60} as ETL.

Sample	V_{oc} (V)	J_{sc} (mA/cm^2)	FF (%)	PCE (%)
1	0.30914	18.53266	69.21244	4.8285
2	0.32247	18.5542	66.3868	4.83665
3	0.32261	20.83649	61.91612	5.06807
4	0.30229	21.32529	63.45465	4.98096
5	0.27583	21.28116	55.11008	3.93916
6	0.32551	20.84809	66.419	5.48848
7	0.25989	21.89855	56.83935	3.93897
8	0.31998	21.47842	66.49982	5.5651
9	0.33248	22.20619	70.10761	6.3028
10	0.26579	22.39516	63.26917	4.58583
11	0.33217	22.22738	69.0288	6.20603
12	0.24687	23.25383	64.83375	4.53206
13	0.32366	23.05362	62.31423	5.66171
14	0.30767	22.76032	63.13277	5.38331
15	0.26604	23.20287	65.68938	4.93752
16	0.33448	22.98025	71.36625	6.67963

17	0.28367	22.38309	67.77377	5.23999
18	0.33297	22.26382	72.54647	6.54865
19	0.31681	18.99516	71.85748	5.2655
20	0.30331	23.47614	67.50762	5.85328
21	0.28023	22.80709	59.72583	4.64809
22	0.31405	22.58581	71.29015	6.15743
23	0.28303	24.03626	64.45477	5.33924
24	0.28002	24.33658	58.42069	4.84787
25	0.29305	21.44536	61.45254	4.70262
26	0.29056	20.06633	62.91257	4.46647
27	0.33699	16.87506	65.9831	4.56907
28	0.30857	22.16223	64.70588	5.38816
29	0.30094	21.89555	62.83032	5.04123
30	0.31504	21.43282	65.7449	5.40553
31	0.30118	21.89266	62.69975	5.03409
32	0.30604	21.4534	66.67445	5.33037
33	0.28558	20.39342	63.59644	4.51011
34	0.31571	20.66282	68.19418	5.41692
35	0.31381	20.23954	69.84699	5.40189
36	0.31704	18.68989	62.80115	4.53124
37	0.33922	18.4176	68.65055	5.22268
38	0.26571	23.01585	65.18015	4.85386
39	0.32407	22.78393	68.36756	6.14687
40	0.28181	22.14826	60.88297	4.62731
41	0.31053	21.97282	63.96589	5.31459
42	0.28432	23.34122	58.68526	4.74237
43	0.299	21.70531	66.32282	5.24116
44	0.31116	21.532	65.50607	5.34421
45	0.29708	22.10698	66.66454	5.33116
46	0.31306	21.92548	66.32933	5.54395
47	0.31178	20.63095	69.60192	5.45148
48	0.31935	20.43196	68.75327	5.46264

Table S6.- Parameters of the TEAI perovskite solar cells with C_{60} as ETL.

Sample	V_{oc} (V)	J_{sc} (mA/cm^2)	FF (%)	PCE (%)
1	0.42163	21.91574	74.90168	6.72008
2	0.45847	21.89548	72.9281	7.1082
3	0.45597	21.97701	67.43385	6.56113
4	0.49189	21.97701	86.7969	8.39361
5	0.483	21.79873	73.16864	7.48004
6	0.46165	21.92018	71.41279	7.01668
7	0.45597	21.97701	67.43385	6.56113
8	0.49189	21.97701	86.7969	8.39361
9	0.483	21.79873	73.16864	7.48004
10	0.46165	21.92018	71.41279	7.01668

11	0.48803	21.86631	82.03108	8.49967
12	0.49189	21.97701	86.7969	8.39361
13	0.49757	21.63713	81.9671	7.74466
14	0.49757	21.63713	81.9671	7.74466
15	0.49757	21.63713	81.9671	7.74466
16	0.4697	23.74629	80.13848	7.01696
17	0.49189	21.97701	86.7969	8.39361
18	0.49189	21.97701	86.7969	8.39361
19	0.49757	21.63713	81.9671	7.74466
20	0.48594	21.79873	84.00635	7.83005
21	0.49757	21.63713	81.9671	7.74466
22	0.45343	21.86631	75.51641	7.90442
23	0.48594	21.79873	84.00635	7.83005
24	0.49189	21.97701	86.7969	8.39361
25	0.49757	21.63713	81.9671	7.74466
26	0.43745	24.05291	75.12087	7.67454
27	0.48594	21.79873	84.00635	7.83005
28	0.42078	24.21996	70.72399	6.99836
29	0.483	21.79873	73.16864	7.48004
30	0.43532	24.45136	71.73399	7.4138
31	0.41217	24.71197	76.05751	7.52184
32	0.41963	23.66794	76.64792	7.39139
33	0.4037	24.52313	73.99226	7.11251
34	0.41183	23.47632	75.43002	7.08092
35	0.40783	24.02772	72.60946	6.90855
36	0.40536	23.82901	71.31444	6.68848
37	0.41568	23.80176	72.87631	7.0009
38	0.483	21.79873	73.16864	7.48004
39	0.42012	23.9761	73.01563	7.14121
40	0.43854	21.86631	82.03108	8.49967
41	0.45847	21.89548	72.9281	7.1082
42	0.46165	21.92018	71.41279	7.01668
43	0.45597	21.86631	82.03108	8.49967
44	0.48803	21.86631	80.92097	8.49967
45	0.45597	23.2385	81.61037	7.1082
46	0.46165	21.92018	71.41279	7.01668
47	0.42163	23.2385	81.61037	7.1082
48	0.483	21.79873	73.16864	7.48004
49	0.483	21.79873	73.16864	7.48004
50	0.46165	21.92018	71.41279	7.01668
51	0.43854	21.86631	82.03108	8.49967
52	0.46165	21.92018	71.41279	7.01668
53	0.48803	21.86631	82.03108	8.49967
54	0.483	21.79873	73.16864	7.48004
55	0.42163	21.91574	74.90168	6.72008
56	0.45597	23.2385	80.92097	7.1082

Table S7.- Parameters of the TEABr perovskite solar cells with C_{60} as ETL.

Sample	V_{oc} (V)	J_{sc} (mA/cm^2)	FF (%)	PCE (%)
1	*0.49757*	*21.63713*	*81.9671*	8.82456715
2	0.54828	23.24775	69.53864	8.86358724
3	0.5522	22.26299	72.61037	8.9264452
4	0.49757	21.63713	75.9671	8.17860794
5	0.55461	23.07015	76.88063	9.83682732
6	0.5622	22.26299	73.61037	9.21326013
7	0.46898	23.47015	76.88063	8.46227474
8	0.45058	25.16948	71.61938	8.1222567
9	0.4744	25.16045	81.61037	9.74110964
10	0.45745	23.95917	80.92097	8.86903729
11	0.54828	23.95326	65.74649	8.63454793
12	0.5322	22.82411	76.88063	9.33868347
13	0.54729	22.81822	71.61938	8.94395968
14	0.53549	22.46315	81.61037	9.81674182
15	0.57461	21.46205	76.88063	9.48115651
16	0.5922	21.49177	69.53864	8.85047908
17	0.55188	22.75646	75.09429	9.43096808
18	0.51729	22.71842	80.92097	9.50984169
19	0.53549	24.45244	65.74649	8.60886979
20	0.5022	24.82411	76.88063	9.58445293
21	0.50729	24.81822	71.61938	9.01690488
22	0.51549	22.46315	81.61037	9.45009662
23	0.57461	20.46205	76.88063	9.03939272
24	0.54828	24.54196	69.12394	9.30122462
25	0.5322	24.49177	75.09429	9.78818024
26	0.55188	22.75646	72.92097	9.15802441
27	0.59729	22.71842	65.74649	8.92146015
28	0.53549	22.45244	73.88063	8.88271033
29	0.48803	22.10681	75.09429	8.10176261
30	0.51495	21.89208	74.41338	8.38886336
31	0.51938	21.98795	70.39848	8.03957785
32	0.49966	20.67654	73.22103	7.56464032
33	0.51495	21.89208	74.41338	8.38886336
34	0.51938	21.98795	70.39848	8.03957785
35	0.46564	22.45262	83.23868	8.70246913
36	0.49966	20.67654	70.0738	7.23949244
37	0.47676	21.89702	73.22103	7.64399968
38	0.51495	21.89208	74.41338	8.38886336
39	0.51938	21.98795	70.39848	8.03957785
40	0.49966	20.67654	78.03495	8.06197795
41	0.47676	21.89702	79.33918	8.28271149
42	0.51495	21.89208	74.41338	8.38886336

43	0.51938	21.98795	70.39848	8.03957785
44	0.46564	22.45262	78.03495	8.15842759
45	0.49966	20.67654	72.67622	7.50835469
46	0.47676	21.89702	83.23868	8.68980459
47	0.51495	21.89208	74.41338	8.38886336
48	0.51938	21.98795	70.39848	8.03957785
49	0.46564	22.45262	78.03495	8.15842759
50	0.49966	20.67654	72.67622	7.50835469
51	0.47676	21.89702	83.23868	8.68980459
52	0.51495	21.89208	74.41338	8.38886336
53	0.47989	21.99713	74.41338	7.85522724
54	0.51938	21.98795	70.39848	8.03957785
55	0.49966	20.67654	72.67622	7.50835469
56	0.47676	21.89702	83.23868	8.68980459
57	0.51495	21.89208	74.41338	8.38886336
58	0.51938	21.98795	70.39848	8.03957785
59	0.46564	22.45262	83.23868	8.70246913
60	0.49966	20.67654	74.41338	7.68782486
61	0.47676	21.89702	70.39848	7.34933609
62	0.51495	21.89208	74.41338	8.38886336
63	0.51938	21.98795	70.39848	8.03957785

Table S8.- Parameters of the TEACl perovskite solar cells with C_{60} as ETL.

Sample	V_{oc} (V)	J_{sc} (mA/cm^2)	FF (%)	PCE (%)
1	0.43414	24.42881	73.22103	7.28645
2	0.40272	24.5365	76.29472	7.07397
3	0.36883	25.16948	69.98908	6.09651
4	0.39389	25.16045	78.57641	7.30689
5	0.3847	23.95917	72.21336	6.2455
6	0.40132	23.95326	78.85062	7.11228
7	0.38457	24.82411	76.07495	6.8147
8	0.39817	24.81822	79.40026	7.36231
9	0.37681	24.46315	74.39619	6.43476
10	0.39428	24.46205	77.41234	7.00574
11	0.40909	24.49177	71.59939	6.73131
12	0.3974	24.91527	74.37997	6.91041
13	0.38965	24.47199	74.32538	6.65017
14	0.39776	23.43895	74.49938	6.5172
15	0.38879	23.99559	73.63608	6.44604
16	0.39421	23.54203	72.67622	6.32869
17	0.43414	24.42881	73.22103	7.28645
18	0.40565	24.8798	78.03495	7.38988
19	0.40565	24.8798	78.03495	7.38988
20	0.42368	23.27788	83.23868	7.7029
21	0.43893	24.54905	68.87093	6.96335

22	0.43414	24.42881	73.22103	7.28645
23	0.43932	23.96585	72.30648	7.14341
24	0.40565	24.8798	78.03495	7.38988
25	0.43893	24.54905	68.87093	6.96335
26	0.43414	24.42881	73.22103	7.28645
27	0.42799	22.85212	67.67466	6.21065
28	0.43414	24.42881	73.22103	7.28645
29	0.40565	24.8798	78.03495	7.38988
30	0.43893	24.54905	68.87093	6.96335
31	0.43414	24.42881	73.22103	7.28645
32	0.43414	24.42881	73.22103	7.28645
33	0.39454	23.86172	72.0747	6.36681
34	0.40565	24.8798	78.03495	7.38988
35	0.39421	23.54203	72.67622	6.32869
36	0.42368	23.27788	83.23868	7.7029
37	0.39347	23.56683	70.0738	6.09701
38	0.43414	24.42881	73.22103	7.28645
39	0.41518	23.991	74.56898	6.96941
40	0.40565	24.8798	78.03495	7.38988
41	0.40421	24.12981	79.33918	7.26097
42	0.43414	24.42881	73.22103	7.28645
43	0.38909	22.20806	77.98591	6.32314
44	0.40565	24.8798	78.03495	7.38988
45	0.40565	24.8798	78.03495	7.38988
46	0.43893	24.54905	68.87093	6.96335
47	0.42368	23.27788	83.23868	7.7029
48	0.43414	24.42881	73.22103	7.28645
49	0.40421	24.12981	79.33918	7.26097
50	0.39507	24.14663	74.06346	6.62962
51	0.398	24.12225	74.6788	6.72751
52	0.39288	24.18244	73.60584	6.56184
53	0.39763	24.16303	74.68905	6.73342
54	0.39284	24.07317	75.11299	6.66517
55	0.40202	24.06127	76.53985	6.94708
56	0.39359	24.54559	75.21088	6.81795
57	0.40272	24.5365	76.29472	7.07397

Table S9.- Parameters of the control perovskite solar cells with ICBA as ETL.

Sample	V_{oc} (V)	J_{sc} (mA/cm^2)	FF (%)	PCE (%)
1	0.64362	20.20351	34.87293	4.53466
2	0.65392	20.09553	46.64671	6.12977
3	0.60754	18.4123	36.73001	4.10867
4	0.64632	18.3203	46.95633	5.56
5	0.63147	18.0044	41.80701	4.75313
6	0.70714	19.17649	48.61126	6.59192
7	0.71229	19.09183	48.26165	6.56305

8	0.70687	18.36557	49.81683	6.46722
9	0.71005	18.27996	49.44235	6.41744
10	0.70149	17.70009	43.22349	5.3668
11	0.70081	17.61198	42.46221	5.24097
12	0.64362	20.20351	34.87293	4.53466
13	0.65392	20.09553	46.64671	6.12977
14	0.60754	18.4123	36.73001	4.10867
15	0.64632	18.3203	46.95633	5.56
16	0.63147	18.0044	41.80701	4.75313
17	0.70714	19.17649	48.61126	6.59192
18	0.71229	19.09183	48.26165	6.56305
19	0.70687	18.36557	49.81683	6.46722
20	0.71005	18.27996	49.44235	6.41744
21	0.70149	17.70009	43.22349	5.3668
22	0.70081	17.61198	42.46221	5.24097
23	0.63147	18.1044	41.80701	4.77313
24	0.71229	19.09183	48.26165	6.56305
25	0.64632	18.3203	46.95633	5.56
26	0.65392	20.09553	46.64671	6.12977
27	0.70714	19.17649	48.61126	6.59192
28	0.71005	18.27996	49.44235	6.41744
29	0.70687	18.36557	49.81683	6.46722
30	0.701229	19.09183	48.26165	6.56305
31	0.70714	19.17649	48.61126	6.59192
32	0.64632	18.3203	46.95633	5.56
33	0.7102	19.09183	48.26165	6.56305
34	0.63147	18.1044	41.80701	4.77313
35	0.6337	18.0044	41.80701	4.75313
36	0.71005	18.27996	49.44235	6.41744
37	0.70714	19.17649	48.61126	6.59192
38	0.70687	18.36557	49.81683	6.46722
39	0.63147	18.1044	41.80701	4.78313
40	0.71229	19.09183	48.26165	6.56305
41	0.70714	19.17649	48.61126	6.59192
42	0.65392	20.09553	46.64671	6.12977
43	0.57379	18.10523	46.28663	4.80778
44	0.57619	18.1254	46.11462	4.81599
45	0.50214	19.32481	38.01978	3.68933

Table S9.- Parameters of the TEABr perovskite solar cells with ICBA as ETL.

Sample	V_{oc} (V)	J_{sc} (mA/cm^2)	FF (%)	PCE (%)
1	0.8017	22.21567	62.71659	11.17005
2	0.80256	23.76707	59.92883	11.43117
3	0.80074	19.99647	60.71292	9.72136
4	0.80459	18.91312	64.64815	9.83769
5	0.78933	21.75525	56.29713	9.66738

6	0.7961	22.01779	56.34745	9.8768
7	0.8017	22.21567	62.71659	11.17005
8	0.80256	23.76707	59.92883	11.43117
9	0.79851	20.68604	58.32301	9.63384
10	0.80024	20.71119	59.0074	9.77988
11	0.78367	22.94892	50.69676	9.1175
12	0.7852	22.91066	54.92255	9.88021
13	0.80119	19.88852	61.271	9.76317
14	0.8038	21.41839	57.71401	9.93614
15	0.8038	21.41839	57.71401	9.93614
16	0.8017	22.21567	62.71659	11.17005
17	0.80256	23.76707	59.92883	11.43117
18	0.80074	19.99647	60.71292	9.72136
19	0.80459	18.91312	64.64815	9.83769
20	0.78933	21.75525	56.29713	9.66738
21	0.7961	22.01779	56.34745	9.8768
22	0.8017	22.21567	62.71659	11.17005
23	0.80256	23.76707	59.92883	11.43117
24	0.79851	20.68604	58.32301	9.63384
25	0.80024	20.71119	59.0074	9.77988
26	0.78367	22.94892	50.69676	9.1175
27	0.7852	22.91066	54.92255	9.88021
28	0.80119	19.88852	61.271	9.76317
29	0.8038	21.41839	57.71401	9.93614
30	0.8041	23.72154	63.30215	11.98
31	0.804	23.70487	63.29651	11.95
32	0.8038	21.41839	57.71401	9.93614
33	0.78175	20.65402	50.3081	8.12289
34	0.78646	21.58209	48.15985	8.17439
35	0.78556	21.01671	47.95554	7.91735
36	0.78756	21.84948	47.32293	8.14323
37	0.79597	22.06535	46.69999	8.20205
38	0.79905	22.02607	50.41242	8.87256
39	0.79597	22.06535	46.69999	8.20205
40	0.79905	22.02607	50.41242	8.87256
41	0.78403	20.10439	51.90252	8.1811
42	0.78175	20.65402	50.3081	8.12289
43	0.78646	21.58209	48.15985	8.17439
44	0.78556	21.01671	47.95554	7.91735
45	0.78756	21.84948	47.32293	8.14323
46	0.80014	20.36757	52.51477	8.55829
47	0.80073	20.34381	53.3679	8.69354
48	0.80074	19.99647	60.71292	9.72136
49	0.80459	18.91312	64.64815	9.83769
50	0.78933	21.75525	56.29713	9.66738
51	0.7961	22.01779	56.34745	9.8768
52	0.78797	20.70741	52.25078	8.52566
53	0.78847	20.67873	53.05472	8.65031

54	0.78756	21.84948	47.32293	8.14323
